# Creeping Wood Sorrel and Chromium Picolinate Effect on the Nutritional Composition and Lipid Oxidative Stability of Broiler Meat

**DOI:** 10.3390/antiox11040780

**Published:** 2022-04-14

**Authors:** Mihaela Saracila, Arabela Elena Untea, Tatiana Dumitra Panaite, Iulia Varzaru, Alexandra Oancea, Raluca Paula Turcu, Petru Alexandru Vlaicu

**Affiliations:** 1 Feed and Food Quality Department, National Research and Development Institute for Biology and Animal Nutrition, Calea Bucuresti, No.1, 077015 Balotesti, Romania; arabela.untea@ibna.ro (A.E.U.); iulia_maros@yahoo.com (I.V.); oancea.alexandra63@yahoo.com (A.O.); raluca.turcu@ibna.ro (R.P.T.); 2 Nutrition Physiology Department, National Research and Development Institute for Biology and Animal Nutrition, Calea Bucuresti, No.1, 077015 Balotesti, Romania; tatiana.panaite@ibna.ro (T.D.P.); alexandru.vlaicu@outlook.com (P.A.V.)

**Keywords:** broiler, chromium picolinate, creeping wood sorrel, meat quality, oxidative stability

## Abstract

The study investigates the efficacy of Cr in broilers, aiming to evaluate the effects of Chromium picolinate (CrPic) in association with creeping wood sorrel powder (CWS) on the proximate composition, fatty acids profile, bioactive nutrients and lipid oxidative stability of broiler meat. A total of 120 Cobb 500 chickens were assigned into three treatments: a control diet (C) and two test diets, including 200 µg/kg diet CrPic (E1), and 200 µg/kg diet CrPic +10 g CWS/kg diet (E2). Dietary supplementation with Cr + CWS significantly improved the concentration of n − 3 polyunsaturated fatty acids (PUFAs), while its n − 6/n − 3 ratio decreased in comparison to the group receiving Cr and the conventional diet. The concentration of docosahexaenoic acid (DHA) significantly increased in the breast meat collected from the E2 group than that from the C group. Dietary administration of Cr and CWS improved lutein and zeaxanthin content, decreased Fe and Zn levels of the breast, and increased Zn deposition in the thigh samples. Malondialdehyde (MDA) concentration decreased more in the thigh meat of the supplemental groups (E1, E2) than in that from the C group. In conclusion, the current study suggests that Cr together with CWS can be a viable option as antioxidant sources for broiler diets, promoting the nutritional quality of meat.

## 1. Introduction

Poultry meat is considered some of the best food protein sources, especially in the developed countries [1]. Enriching the nutritional quality of chicken is a hotly debated field of research, especially in the current conditions in which some foods are seen as food-medicine. Fatty acids, mainly long chain polyunsaturated fatty acids (LC-PUFAs), carotenoids, polyphenols, minerals and vitamins, are the most studied nutrients suitable for meat enrichment. However, enriching meat with nutrients such as fatty acids can also bring some disadvantages, such as accelerating lipid oxidation, which negatively affects their quality and shelf life.

Feed has been described as one major factor influencing the resultant chemical composition of edible products from poultry. Thus, the most common strategy for manipulating the nutritional composition of poultry remains feed supplementation.

Chromium is not currently considered an essential trace element for poultry, but it is thought that this micronutrient may play a nutritional and physiological role. Dietary Cr supplementation has been reported to have a positive effect on meat quality of broiler chicks in natural [2] or heat stress conditions [3,4]. In addition, Cr can improve the metabolism of carbohydrates and lipids, reduce stress reactions, and stimulate the immune and antioxidant system [5].

Bioactive compounds of herbs can prevent the oxidative stress which play a significant role in the health of poultry and therefore in the quality of meat.

Creeping wood sorrel (CWS) is a well-known medicinal plant with versatile nutritional uses. Regarding the proximate chemical composition, CWS contains a low amount of crude protein and crude fat [6]. Phytochemical composition of CWS showed the presence of niacin, vitamin C and β-carotene, neutral lipids, glyoxylic acid, pyruvic acid, oxalic acid, vitexin and glycolipids [7,8].

In studies in heat-stressed chicks, it has been postulated that CWS and Cr picolinate (CrPic) may have a synergistic action [3,4] in combating the effects of temperature on some parameters, such as performance and meat quality. The association of CrPic with CWS in broiler diet improved the textural parameters [3] and oxidative stability of meat [4]. However, no studies have been performed to investigate their effect under thermoneutral conditions. Despite the extensive investigation of Cr and CWS individuals, scarce data are available on their combined effect on meat composition, especially on fatty acid profiles in broilers. Therefore, in order to investigate the efficacy of CrPic in association with CWS in broiler diets, the present study aimed to evaluate their effects on the proximate composition, fatty acids profile, bioactive nutrients and lipid oxidative stability of broiler meat.

## 2. Materials and Methods

### 2.1. Birds and Experimental Design

The 28-day feeding trial was performed on 90-day-old chicks of the Cobb 500 in an experimental hall according to the experimental protocol approved by the Ethical Commission of National Research and Development Institute for Biology and Animal Nutrition. Until 14 days of age, the chicks were fed a conventional basal diet. On the 14th day, the chicks were randomly assigned to 3 treatment groups, 30 chicks per treatment, and 5 replicates with 6 birds each. The chicks were housed in three-tiered digestibility cages (65 × 75 × 45 cm) and reared under controlled environmental conditions. In the first 3 days, a temperature of 34 °C was ensured, after which it was gradually reduced until the thermal comfort temperature of 26 °C was reached. All cages were equipped with feeders and waterers. The treatments consisted of a corn and soybean meal-based control diet with no Chromium addition (C), and two test diets in which the basal diet was supplemented with either 200 µg/kg diet chromium (E1), 200 µg/kg diet chromium plus 10 g creeping wood sorrel powder (CWS)/kg diet (E2). Chromium picolinate (CrPic, Santa Cruz Biotechnology, Santa Cruz, CA, USA) was used as a Cr source. The creeping wood sorrel was harvested in their late vegetative stage (44.62° N, 26.12° E). Ingredients and chemical composition of the basal diet are shown in Table 1. Feed and water were available ad libitum. Temperature and air relative humidity were recorded daily throughout the experimental period using a Viper Touch computer. The light regimen was 23 h light/1 h darkness. The chicks were vaccinated, after which no medical care program or treatment was applied.

### 2.2. Meat Samples Collection

At 42 days, 6 chickens/group were randomly selected, electrically stunned, and slaughtered by cervical dislocation. After bleeding and evisceration, thigh and breast meat samples were collected (6 breast and 6 thigh meat samples per group). The samples collected were used to determine the proximate composition (dry matter, DM; crude protein, CP; ether extractives, EE; ash), fatty acids profile, bioactive nutrients and end products of lipid oxidation (TBARS).

### 2.3. Extraction Technique for Total Phenol Content (TPC) and Total Antioxidant Capacity (TAC) Analysis of Samples

The extraction of total phenols from plant and feed samples was carried out using methanol 80% p.a., (1:10, *w*/*v*) and kept on a rotary shaker for 24 h in a dark place. The extract was centrifuged for 10 min at 1500× *g*. The supernatant collected was used for further analysis.

The breast and thigh meat samples were extracted using a solution of phosphate-buffered saline (PBS), pH 7.4 (1:10, *w*/*v*). The samples were centrifugated (10,000× *g* at 4 °C, for 30 min). After the centrifugation, the supernatant was taken and used for further analysis.

### 2.4. Extraction Technique for Liposoluble Compounds Analysis of Samples

The extraction of liposoluble compounds was performed according to the method previously described by [9], and included a saponification step and an extraction procedure with petroleum ether and ethanol.

### 2.5. Chemical Composition Analysis

The proximate composition (dry matter, DM; crude protein, CP; ether extract, EE; and ash) of samples was determined according to the chemical methods described previously [3].

The total phenol content (TPC) was measured spectrophotometrically according to the Folin–Ciocalteu method, described by [10], using a V-530 Jasco (Japan Servo Co., Ltd., Tokyo, Japan) spectrophotometer. The results were expressed as mg gallic acid equivalent (GAE)/g.

The total antioxidant capacity (TAC) was evaluated by the DPPH method described previously by [10] using a V-530 Jasco (Japan Servo Co., Ltd., Tokyo, Japan) spectrophotometer. The values were expressed as mmol Trolox equivalents /kg sample.

Lutein and zeaxanthin content of samples were analysed using the method described previously [9], with a high-performance liquid chromatograph HPLC series 200 (Perkin Elmer, Shelton, CT, USA) and a Nucleodur C18 column (Macherey-Nagel, Germany).

Vitamin E determination (expressed as mg/kg) was performed according to the method described by [9], using a high-performance liquid chromatograph (HPLC Finningan Surveyor Plus, Thermo-Electron Corporation, Waltham, MA, USA), and a PDA-UV detector at a wavelength of 292 nm.

Trace mineral (Cu, Fe, Mn, Zn) concentrations (expressed as mg/kg) were determined by flame atomic absorption spectrometry (FAAS) using a Thermo Electron—SOLAAR M6 Dual Zeeman Comfort (Cambridge, UK) equipment after microwave digestion, according to [11].

The fatty acid content of breast and thigh samples was determined by gas chromatography using a PerkinElmer Clarus 500 (Waltham, MA, USA) equipped with a flame ionization detector (FID) and capillary separation column with a high polar stationary phase TRACE TR-Fame, (Thermo Electron, Waltham, MA, USA), according to [12]. The results were expressed as g/100 g fatty acids methyl esters (FAME).

Nutritional quality indices of meat lipids were assessed by calculating the atherogenicity index (IA), thrombogenicity index (IT), the ratio of hypocholesterolemic and hypercholesteremic fatty acids (h/H), and the PUFA n − 6/PUFA n − 3, Σ SFA/Σ UFA, Σ PUFA/Σ MUFA ratios. Indices of atherogenicity (AI), thrombogenicity (TI) and saturation (SI), and the ratio of hypo and hypercholesterolemia (h/H), were calculated according to [13,14] as follows:

AI = C12:0 + (4 × C14:0) + C16:0]/ΣUFA(1)

TI = (C 14:0 + C16:0 + C18:0)/[(0.5 × MUFA) + (0.5 × Σ n − 6) + (3 × Σ n − 3) + (Σ n − 3/Σ n − 6)](2)

SI = C 14:0 + C 16:0 + C 18:0/MUFA + PUFA(3)

h/H = [(C18:1 + C18:2 + C18:3 + C20:3 + C20:4 + C20:5 + C22:4 + C22:5 + C22:6)/(C14:0 + C16:0)](4)

The health-promoting index (HPI) assess the nutritional value of dietary fat, and was calculated according to Chen et al. (2004) using the following formula:

HPI = ΣUFA/[C12:0 + (4 × C14:0) + C16:0](5)

The lipid oxidative stability of broiler meat was evaluated using the TBARS (thiobarbituric acid reactive substances) method using third derivative spectrophotometry, which measures the end products of lipid oxidation. TBARS were determined according to the method described by [13]. The substances which react with thiobarbituric acid were expressed as µg malondialdehyde (MDA) per kilogram sample (µg MDA/kg^−1^). The calibration curve was performed (1,1,3,3-tetramethoxypropane hydrolysed to MDA stock solution) by plotting MDA known concentrations (working solutions) versus absorbance of analytical signals. For the MDA extraction in real samples, a mixture of TCA-BHT (2:1, *v*:*v*) was used, and a 50 min (80 °C) incubation time. The third derivative spectrum (540 nm) of pink intensity of reaction was recorded and converted into MDA concentration values.

### 2.6. Statistical Analysis

The data were analyzed by one-way analysis of variance (ANOVA), and the means were compared applying Tukey’s multiple range test using IBM SPSS Statistics (version 27.0 for windows, SPSS Inc., Chicago, IL). Differences were considered statistically significant at *p* < 0.05 unless otherwise stated. Graphs were drawn using Prism-GraphPad software v. 9.02 (San Diego, CA, USA). Correlation between bioactive nutrients and lipid oxidative stability were analyzed by Pearson’s correlation coefficient test and displayed as a correlation heatmap. The heatmap is symmetric along the diagonal. Blue colors correspond to positive correlation coefficients, red colors correspond to negative correlation coefficients. The saturation of colors reflects the absolute value of the correlation coefficient. The significant correlations are marked with: * *p* < 0.05 if the correlation is significant at alpha = 0.05 level; ** *p* < 0.01 if the correlation is significant at alpha = 0.01 level; *** *p* < 0.001 if the correlation is significant at alpha = 0.001 level.

## 3. Results

### 3.1. Chemical Composition of Creeping Wood Sorrel (CWS)

Table 2 presents the chemical composition of dietary creeping wood sorrel used in the diet formulation. The proximate composition revealed important concentrations of crude protein, crude fiber and ash.

The creeping wood sorrel showed relatively low concentration of polyphenols, but high concentrations of lutein and zeaxanthin and vitamin E, the latter resulting in a high total antioxidant capacity.

CWS did not contain significant amounts of fatty acids, not being a fat source. Thus, from the analysis of feed compounds, no differences were observed between diets in terms of the content of fatty acids. The mineral profile reveals significant levels of Fe and Zn and low levels of Cu andMn.

### 3.2. Proximate Composition of Meat

Table 3 shows the proximate composition of the breast and thigh meat collected.

The dietary association of Cr and CWS lowered the EE content of breast meat compared to the control and E1 diet. Instead, chromium supplemented alone had no significant effect on EE content in breast meat. The diet supplementation had no influence on the proximate composition of thigh meat.

### 3.3. Fatty Acid Profile of Broiler Meat

The effects of experimental treatments on fatty acid compositions in the breast muscle of broiler chickens are presented in Table 4. The use of Cr alone or in association with CWS led to a significant (*p* < 0.05) alteration of some of the fatty-acid profile of breast meat.

In the SFAs family, statistically significant differences were found for C15:0 acid: C > E1 = E2, C16:0 acid: E2 > C = E1; C17:0 acid: E2 < E1 = C; C18:0 acid: E1 = C < E2; C24:0 acid: E1 < C = E2. Total SFAs, PUFAs and PUFA n − 6 did not differ between groups. In the MUFAs family, statistically significant differences were found for C14:1 acid: E1 = E2 < C; C16:1 acid: E2 < C = E1; C17:1 acid: E1 = E2 > C; C18:1 acid: E2 < E1 = C; C22:1n9 acid: E2 < E1 = C; C24:1n9 acid: E1 < C = E2. In the E2 group, lower MUFAs (*p* = 0.009) were found compared to the C and E1 group. The following relationships were noted in the n − 6 fatty acid family: C18:2n6 acid—E2 < C = E1; C18:3n6 acid: E2 < C = E1; C20:3n6 acid: E1 < E2 = C; C20:4n6 acid: E2 > E1 = C; C22:2n6 acid: E2 > E1 = C; C22:4n6 acid: E2 > E1 = C.

A higher content (*p* = 0.007) of n − 3 PUFAs was measured in the breast meat of chickens from the E2 group compared to the other two groups. In the n − 3 fatty-acids family, statistically significant differences were found for α-linolenic acid (C18:3n3): C > E1 = E2; C18:4n3 acid: E2 > E1 > C; C20:3n3 acid: E2 > E1 = C; C20:5n3 acid: E2 = E1 < C; C22:6n3 acid: E1 = E2 > C.

Table 5 shows the fatty-acid profile of thigh meat. Total SFAs, PUFAs, PUFA n − 6 and PUFA n − 3 did not differ between groups. In the SFAs family, statistically significant differences were found for C18:0 acid: C < E1 = E2. A lower content (*p* = 0.012) of MUFAs was measured in the thigh meat of chickens from the E2 group compared to the C group. In the MUFAs family, statistically significant differences were found for C16:1 acid: C > E1 = E2; C24:1n9 acid: E2 = E1 > C. The following relationships were noted in the n − 6 fatty acid family: C20:2n6 acid: E1 = E2 < C; C22:3n6 acid: E1 > C = E2. In the n − 3 fatty-acids family, statistically significant differences were found for C18:3n3 acid: E1 = E2 < C; and C18:4n3 acid: E1 = E2 < C.

### 3.4. Nutritional Quality Indices of Meat Lipids

**Figure 1:**
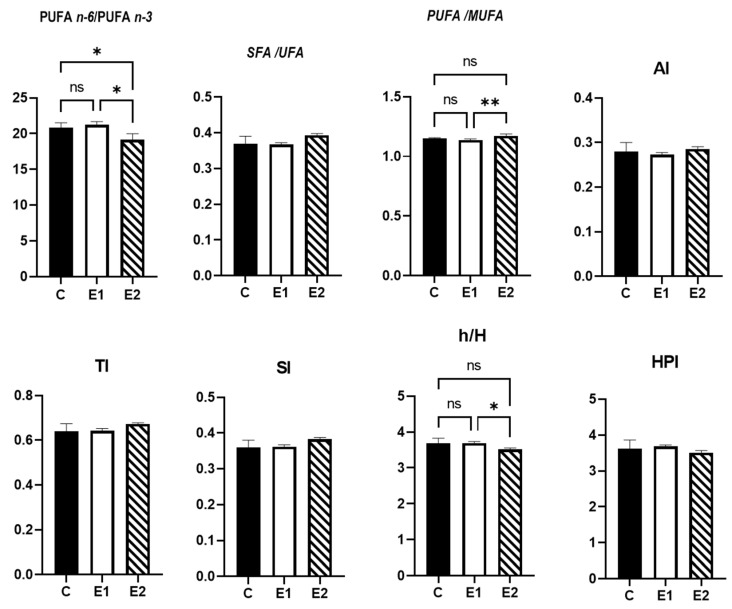
Nutritional quality indices of the lipids in breast meat. Main effects of diet are presented in each graph (Prism Graph 9.02). Data are presented as mean SEM (*n* = 6 broilers/group). Asterisks denote statistical significance (*p* > 0.1234 ns; * *p* ≤ 0.0332; ** *p* ≤ 0.0021); AI = atherogenic index; TI = thrombogenicity index; SI = saturation index; h/H = hypo/hypercholesterolemic index; HPI = health-promoting index; C—control diet; E1 = experimental diet supplemented with 200 µg/kg diet CrPic; E2 = experimental diet supplemented with 200 µg/kg diet CrPic + 10 g creeping wood sorrel powder (CWS)/kg diet.

The lowest n − 6/n − 3 ratio was observed in the E2 group (Figure 1).

The PUFA/MUFA ratio was significantly higher in the breast from the E2 group than in those from the E1 group. However, the h/H ratio was significantly lower in the breast from E2 than those collected from the E1 group. The SFA/UFA ratio, AI, TI, SI, and HPI value did not record significant differences between groups.

**Figure 2:**
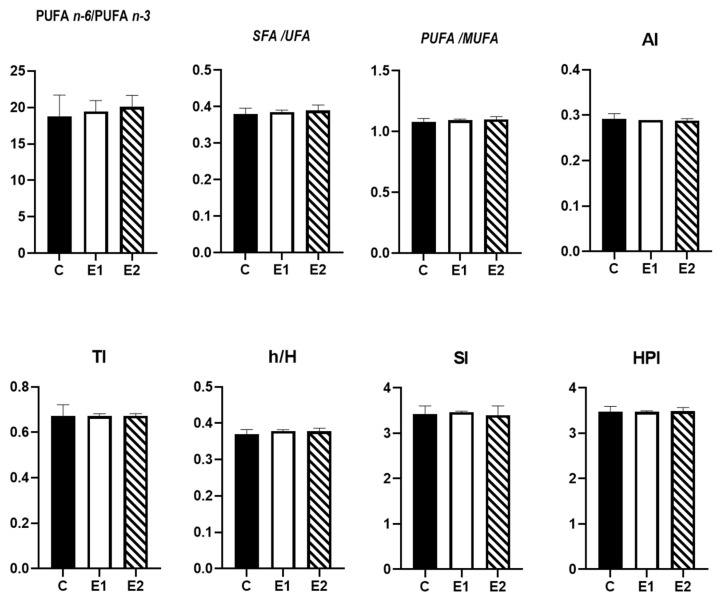
Nutritional quality indices of the lipids in thigh meat. Main effects of diet are presented in each graph (Prism Graph 9.02). Data are presented as mean SEM (*n* = 6 broilers/group); AI = atherogenic index; TI = thrombogenicity index; SI = saturation index; h/H = hypo/hypercholesterolemic index; HPI = health-promoting index; C—control diet; E1 = experimental diet supplemented with 200 µg/kg diet CrPic; E2 = experimental diet supplemented with 200 µg/kg diet CrPic + 10 g creeping wood sorrel powder (CWS)/kg diet.

Regarding the nutritional quality indices (Figure 2), PUFA n − 6/n − 3, SFA/UFA, PUFA/MUFA ratio, AI, TI, h/H, SI and HPI values did not record significant differences between groups.

### 3.5. Bioactive Nutrient Content of Breast and Thigh Meat

Table 6 shows the bioactive nutrient content of breast meat. The TPC and TAC did not show significant differences between groups.

Lutein and zeaxanthin content of the breast from E2 was significantly higher than in group C. The vitamin E level was significantly lower in E1 and E2 groups than in the C group. The level of Fe significantly decreased in the breast samples from groups fed Cr and Cr + CWS compared to those fed the C diet. Zn level decreased only in the breast collected from the E1 group vs. C. Cu and Mn levels were not detected in the breast samples.

Table 7 shows the bioactive nutrient content of thigh meat. The TPC and TAC parameters did not show significant differences between groups. Lutein and zeaxanthin content of the breast from E2 was significantly higher than in group C. The vitamin E level was significantly lower in E2 group than in the C group. The thigh samples from the E1 group had a significantly higher level of Fe compared to those from the C and E1 group. The Zn level in the thigh samples were significantly higher in the E2 group than in the C and E1 group. Cu and Mn levels did not record any significant difference between groups.

### 3.6. Oxidative Stability of Broiler Meat

**Figure 3:**
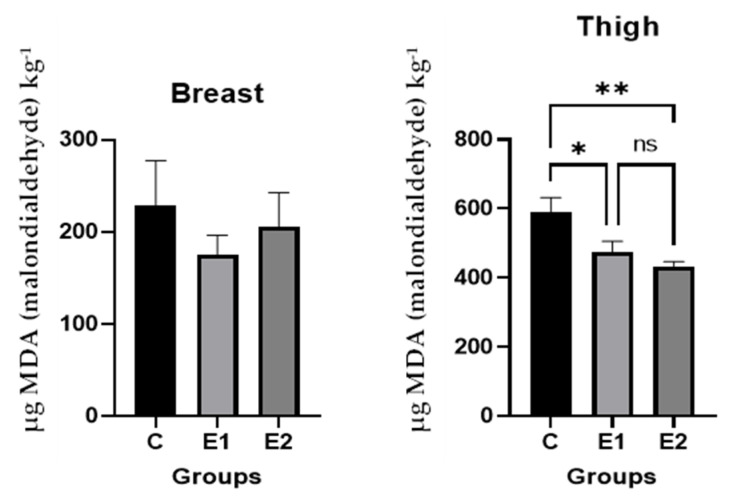
TBA reactive substances (TBARS) of broiler breast and thigh meat during seven days of storage. Main effects of diet are presented in each graph (Prism Graph 9.02). Data are presented as mean SEM (*n* = 6 broilers/group). Asterisks denote statistical significance (*p* > 0.1234 ns, * *p* ≤ 0.0332; ** *p* ≤ 0.0021); C—control diet; E1 = experimental diet supplemented with 200 µg/kg diet CrPic; E2 = experimental diet supplemented with 200 µg/kg diet CrPic + 10 g creeping wood sorrel powder (CWS)/kg diet.

The effect of feeding Cr alone or in combination with CWS on TBARS of broiler breast and thigh meat during seven days of storage is depicted in Figure 3. Incorporation of Cr and Cr + CWS in the bird′s diet led to significant reductions in TBARS of thigh meat during seven days of refrigeration storage. The TBARS of thigh meat from E1 and E2 groups were reduced up to 19.58% or 26.65% during seven days of storage, respectively, when compared with the control (*p* < 0.05). However, no effect of diet on TBARS of breast meat at seven days of storage was observed among treatment groups.

**Figure 4:**
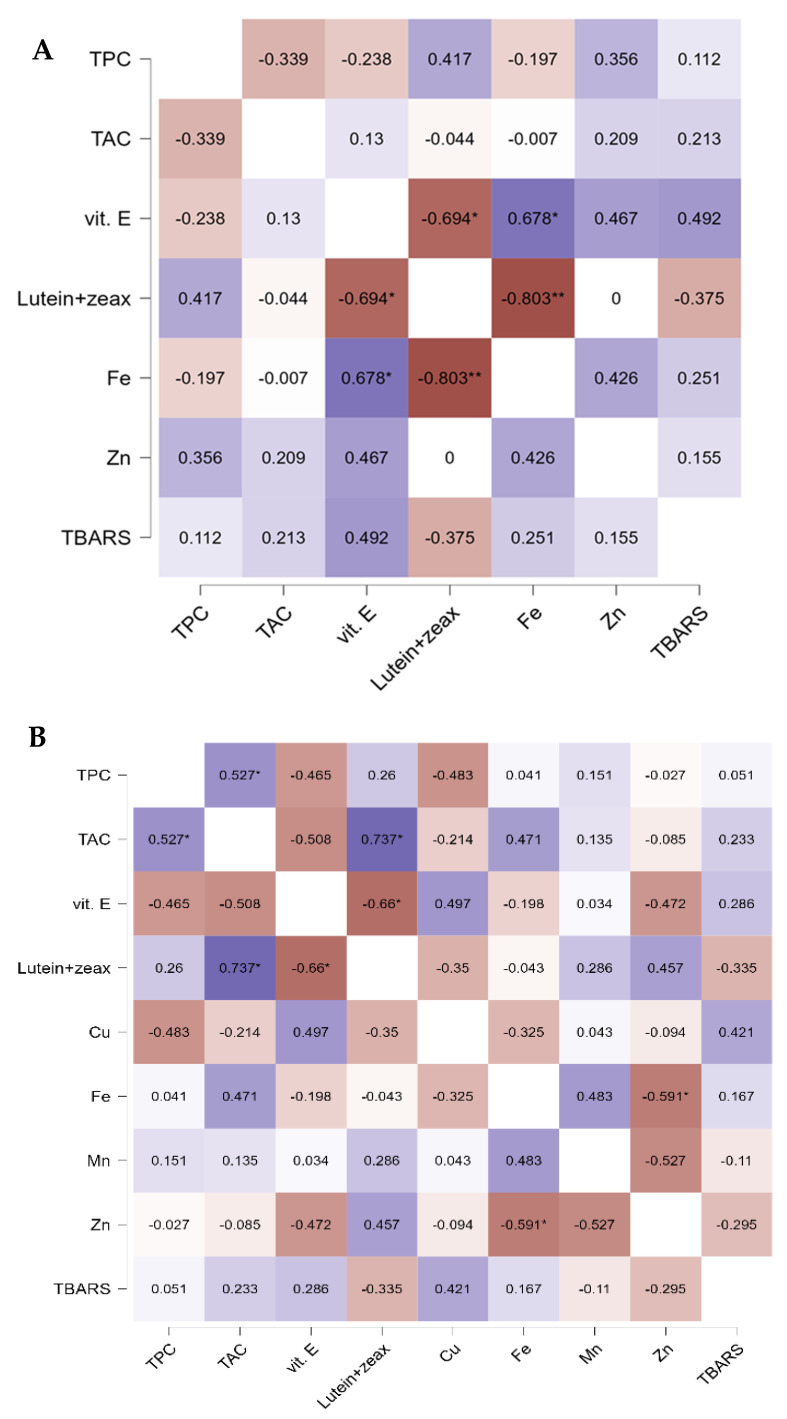
Heat map showing Pearson’s correlations between bioactive nutrients content, TAC and TBARS in breast (**A**) and thigh meat (**B**). Abbreviations: TPC, total phenolic content; TAC, total antioxidant capacity; TBARS, thiobarbituric reactive substances. Blue colors correspond to positive correlation coefficients, red colors correspond to negative correlation coefficients. The saturation of colors reflects the absolute value of the correlation coefficient. Asterisks indicate a significant correlation between respective parameters: * *p* < 0.05 if the correlation is significant at alpha = 0.05 level; ** *p* < 0.01 if the correlation is significant at alpha = 0.01 level; *p* < 0.001 if the correlation is significant at alpha = 0.001 level.

Figure 4 shows the correlation coefficients between bioactive nutrients (TPC, vit. E, lutein and zeaxanthin, Fe, Cu, Mn, Zn), TAC and TBARS in breast (A) and thigh (B) meat samples. In the breast samples (Figure 4A), vitamin E was negatively correlated with lutein and zeaxanthin (*r* = −0.694, *p* < 0.05) and positively correlated with Fe (*r* = 0.678, *p* < 0.05). In addition, Fe and lutein and zeaxanthin content were strongly negative correlated (*r* = −0.803, *p* < 0.01).

In thigh samples (Figure 4B), TAC was positively correlated with TPC (*r* = 0.527, *p* < 0.05) and lutein and zeaxanthin (*r* = 0.737, *p* < 0.05). Vitamin E was negatively correlated with lutein and zeaxanthin (*r* = −0.66, *p* < 0.05). Regression analysis showed that the correlations were negatively significant (*r* = −0.591, *p* < 0.05), regardless of Fe and Zn content in the thigh samples.

## 4. Discussion

The characterisation of CWS revealed important concentrations of crude protein, crude fiber, ash and high concentration of lutein and zeaxanthin and vitamin E, making it a valuable natural supplement.

The combination of different sources of antioxidants administered in the diet of chickens has been reported to positively influence the proximate composition of broiler meat. In our study, the administration of Cr + CWS in the broiler’s diet decreased the crude fat content in breast meat, as other authors concluded in previous studies [15,16]; however, in other reports [17] it was observed that Cr supplemented diets did not affect crude fat composition of broiler meat (33 °C). A possible explanation might be the synergistic action of chromium and other antioxidants in modifying the lipid profile of carcass [18], or that Cr can influence the fat deposition in tissues by decreasing the enzymatic activities of fatty acid synthase, acetyl-CoA carboxylase, hormone-sensitive lipase, and lipoprotein lipase [19].

Feed has been described as one major factor influencing the resultant lipid and FA composition of edible products from poultry [20]. The nutritional value of chicken meat is determined, among other nutrients, by its fatty acid content, and can be a determining factor in its storage or further processing [21]. From the consumer′s point of view, enriching meat with n − 3 PUFA or the balance of n − 6/n − 3 PUFA in broilers is important [22]. In the present study, the breast meat of chickens receiving Cr + CWS contained significantly higher n − 3 PUFAs, while its n − 6/n − 3 ratio was lower in comparison to the group receiving Cr and conventional diet. Those achieves are of interest in the nutritional quality of poultry meat for human health. To the best of our knowledge, the available information on broiler tissues is limited to the effect of Cr supplementation alone or in association with other ingredients on the fatty acid profiles of broilers. In line with our results, other authors [23] reported that dietary antioxidant sources (vitamin E or sweet chestnut tannins) in broiler diets increased total PUFA and omega-3, especially EPA and DHA. Previous studies carried out by our team [16] did not show any significant impact of Cr (200 and 400 µg/kg of feed material) in association with 3% camelina meal on n − 3 PUFAs, SFA, MUFA and PUFA totals, despite differences in the content of certain fatty acids found between groups.

In the present study, α-linolenic acid (ALA, C18:3n3) was found in a significantly lower concentration in the breast meat from experimental groups than in the C group. Over time, much attention has been paid to meat enrichment in ALA, as it is known to be the main precursor for long chain polyunsaturated fatty acids (LC-PUFAs), such as eicosapentaenoic acid (EPA, C20:5n3) and docosahexaenoic acid (DHA, C22:6n3). Recent findings have shown that it is not the concentration of ALA that is important, but rather the conversion rate to the aforementioned LC-PUFAs, especially DHA. In humans, elevating EPA and DHA intakes strongly reduced cardiovascular morbidity [24]. Our body can convert EPA to DHA to a limited extent. DHA synthesis rates from ALA are suggested to be low relative to dietary intake and tissue demand [25]. DHA cannot be synthesized de novo in mammals, and therefore must be obtained in the diet primarily through fish, nutraceuticals and functional foods [26], or synthesized within the body from ALA. In our study, DHA concentration significantly increased in the breast meat collected from groups fed diets supplemented with Cr + CWS (E2), compared to those from the C group. Thus, the diet supplemented with Cr and CWS led to an effectiveness in converting ALA to DHA and its deposition in the pectoralis muscle. Some authors [27] showed that the concentration of DHA in meat samples were increased in 3 g/kg sweet chestnut tannins and 200 mg/kg α-tocopherol groups. In the thigh samples, supplementation of Cr in combination with CWS had an effect only in lowering the total content of MUFAs.

Regarding the nutritional quality indices of the lipids, the lowest n − 6/n − 3 ratio was observed in the group fed the Cr + CWS diet. In our study, PUFA/MUFA and h/H ratios were significantly higher in the breast from the E2 group than in those from the E1 group. The h/H ratio illustrates the effect of fatty acids on cholesterol metabolism, so a healthy meat should have high values [28]. This evidence demonstrates that the association of Cr with CWS had a greater effect in promoting nutritional quality of lipids from breast meat than Cr alone. A possible explanation for the improved de novo synthesis might be that chromium provides more energy for synthesis by improving the glucose utilization [29].

Meat and meat-based products are an excellent source of bioactive compounds with beneficial health effects, such as vitamins, minerals, peptides or fatty acids [30]. Diet supplementation with antioxidant sources (natural or synthetic) could represent an excellent way to improve the content of meat in bioactive nutrients. Results from the present study revealed that the dietary administration of Cr and CWS improved lutein and zeaxanthin content of the breast more than the conventional diet. This achievement is important due to the role of these nutrients in visual function, retina protection, free radical scavenging, etc. [31], and it has a positive effect on consumer perception, with xanthophyll being associated with more healthy yellow products. Our findings showed that the vitamin E level was significantly lower in E1 and E2 groups than in the C group. This may be due to increased levels of lutein and zeaxanthin, with an antagonistic effect between lutein and zeaxanthin and vitamin E, as evidenced by existing studies in the scientific literature [32,33]. However, as far as we know, there have been no studies to demonstrate this aspect in poultry yet. However, the underlying mechanism remains unclear and requires further research.

The present findings showed that Cr supplementation decrease the Zn deposition in the thigh samples, but in association with CWS, increases it. In line with this, several authors [34,35] found that birds supplemented with dietary Cr had a decreased retention of Zn in leg muscles. However, the levels of Fe significantly decreased in the breast samples from the groups fed Cr and Cr + CWS compared to those fed the C diet. This impairment in Fe deposition can be explained by the antagonism between Cr and Fe, since the minerals are competitors for the same binding sites [36].

Lipid oxidation is a spontaneous process that takes place postharvest in meat and is augmented by endogenous (total lipids, amount of Fe present, and fatty acid composition) and exogenous factors including heating, disruption of membranes, or prolonged storage [37]. Concentration of thiobarbituric acid reactive substances (TBARS) was measured as a marker of oxidative damage, being the main biomarker of lipid peroxidation. In this study, the administration of Cr alone (E1) or in association with CWS (E2) delayed the oxidation processes observed by decreasing the concentration of MDA in thigh meat samples, compared with the C diet. However, it should be pointed out that the experimental diets did not affect the oxidative stability in the breast muscle when compared with the control diet. The thigh meat is a matrix with higher lipid content than breast tissue, and it is more susceptible to the occurrence of oxidative processes. The effectiveness of the association of Cr and CWS in preventing lipid peroxidation through postharvest application in refrigerated broiler meat has been previously reported [4]. The same authors showed in a previous study [16] that dietary Cr supplementation has a positive effect on lipid peroxidation of broiler meat. Under heat stress conditions, dietary Cr nicotinate and Cr chloride (500; 1000; and 1500 μg/kg) enhanced oxidative stability of refrigerated breast and thigh meat over two or six days of storage time [38]. According to the antioxidant theory, when the concentrations of antioxidants decrease, lipid peroxidation increases in plasma and tissues, leading to damage of cell membranes. In the present study, and parallel to this theory, the high concentration of antioxidants (Cr and CWS) decreases lipid peroxidation. This claim is confirmed by similar reports in which herbs and spices in combination with Cr even more effectively prevent lipid peroxidation in the tissues of broilers [4,39].

Our study reveals that there exists negative correlation between vitamin E and lutein and zeaxanthin in the breast and thigh samples, observation statistically sustained by the heat map (Figure 4). This may be due to the fact that vitamin E competes for absorption with other lipid micronutrients (e.g., carotenoids) as they have the same site of absorption (in the upper half of the small intestine) [40]. In a study [41], carotenoid supplementation decreased the vitamin E level in leg samples. However, the literature is scarce in studies evidencing this relationship. In addition, Fe was strongly negatively correlated with lutein and zeaxanthin content (*r* = −0.803, *p* < 0.01), and positively correlated with vitamin E (*r* = 0.678, *p* < 0.05). Vitamin E is the most potent liposoluble antioxidant and has the potential to improve tolerance of Fe supplementation [42].

Pearson’s correlations in the thigh samples (Figure 4) showed a positive correlation between TAC and TPC (*r* = 0.527, *p* < 0.05), and TAC and lutein and zeaxanthin (*r* = 0.737, *p* < 0.05), which means that polyphenols and xanthophylls are the major contributors to the total antioxidant capacity. The negative correlation between Fe and Zn content in the thigh samples (Figure 4) is due to the aforementioned competition for the same transferrin binding sites. In contrast, there are studies in the literature that have revealed a positive correlation between Zn and Fe in pork [43].

## 5. Conclusions

Early studies reported the antioxidant potential of Cr and CWS supplements to broiler diets, but its efficacy on nutritional quality of meat was not investigated. In conclusion, the current study suggests that Cr together with CWS can be a viable option as antioxidant sources for broiler diets, promoting the nutritional quality of meat in terms of the positive effect on lipid quality (increased n − 3 PUFA and converting potential of ALA to DHA) and lipid stability (reduced lipid peroxidation process); and improving the bioactive nutrients deposition (xanthophylls and minerals) in particular tissues. Finding out the details of the relationship between Cr and CWS would appreciate attention in future research, and the results of this study indicate that this relationship is a crucial one. The mechanism by which Cr and CWS can convert ALA to the desirable fatty acid, DHA, requires further research.

## Figures and Tables

**Table 1:** Nutrient composition of experimental basal diets (%).

Ingredient	Grower (14–28 d)			Finisher (28–42 d)		
	C	E1	E2	C	E1	E2
	%					
Corn	40.18	40.18	39.28	44.70	44.70	43.70
Soybean meal	26.33	26.33	26.20	21.32	21.32	21.32
Wheat	20.00	20.00	20.00	20.00	20.00	20.00
Corn gluten	5.00	5.00	5.00	5.00	5.00	5.00
Creeping wood sorrel (CWS)	-	-	1.00	-	-	1.00
Oil	3.78	3.78	3.80	4.62	4.62	4.62
Monocalcium phosphate	1.36	1.36	1.36	1.19	1.19	1.19
Calcium carbonate	1.25	1.25	1.24	1.13	1.13	1.13
Salt	0.36	0.36	0.36	0.36	0.36	0.36
Methionine	0.30	0.30	0.3	0.26	0.26	0.26
Lysine	0.30	0.30	0.31	0.30	0.30	0.30
Treonine	0.09	0.09	0.10	0.07	0.07	0.07
Choline	0.05	0.05	0.05	0.05	0.05	0.05
A1 Premix	1.00 ^1^	1.00 ^2^	1.00 ^3^	1.00 ^1^	1.00 ^2^	1.00 ^3^
Total	100	100	100	100	100	100
Chemical analysis—theoretical						
Metabolisabe energy, Kcal/kg	3128.99			3217.72		
Crude protein, %	21.50			20.00		
Ether extract, %	6.01			6.49		
Crude fiber, %	3.57			3.36		
Ca., %	0.87			0.81		
P, %	0.70			0.65		
Available phosphorus., %	0.43			0.41		
Antioxidant profile—analysed						
TPC, mg/g GAE	1.70	1.75	1.99	1.83	1.84	1.97
TAC, mmol Trolox equivalents/kg	5.70	6.08	6.72	6.00	6.27	7.12
Lutein and zeaxanthin, µg/g	10.11	10.02	10.43	9.90	9.14	9.47
Vitamin E, µg/g	40.13	37.92	40.01	40.45	38.19	39.92

^1^ 1 kg of A1 premix contains 1,100,000 IU/kg vitamin A; 200,000 IU/kg vitamin D3; 2700 IU/kg vitamin E; 300 mg/kg vitamin K; 200 mg/kg Vit. B1; 400 mg/kg vitamin B2; 1485 mg/kg pantothenic acid; 2700 mg/kg nicotinic acid; 300 mg/kg vitamin B6; 4 mg/kg Vit. B7; 100 mg/kg vitamin B9; 1.8 mg/kg vitamin B12; 2000 mg/kg vitamin C; 8000 mg/kg manganese; 8000 mg/kg iron; 500 mg/kg copper; 6000 mg/kg zinc; 37 mg/kg cobalt; 152 mg/kg iodine; 18 mg/kg selenium. C: control diet; E1: experimental diet supplemented with 200 µg/kg diet CrPic; E2: experimental diet supplemented with 200 µg/kg diet CrPic + 10 g creeping wood sorrel powder (CWS)/kg diet. ^2^ A1 premix + 20 mg CrPic/kg premix; ^3^ A1 premix + 20 mg CrPic/kg premix + 1% creeping wood sorrel powder; TPC- total phenolic content; TAC- total antioxidant capacity.

**Table 2:** Chemical composition of creeping wood sorrel (CWS).

Analysed Parameters	Creeping Wood Sorrel (CWS)
Proximate composition (%)	
DM	92.18
CP	16.74
EE	3.18
CF	19.36
Ash	22.56
Fatty acids (g/100 g FAME)	
C18:3n3	0.98
SFA	36.35
MUFA	43.54
PUFA	19.55
UFA	63.09
Σ n − 3	2.98
Σ n − 6	15.73
Σ n − 6/n − 3	5.28
Antioxidant profile	
TPC, mg/g GAE	7.87
TAC, µM Trolox	170.70
Lutein and zeaxanthin, µg/g	267.41
Vitamin E, µg/g	210.93
Mineral profile (mg/kg)	
Copper	6.40
Iron	243.68
Manganese	41.00
Zinc	92.10

DM—dry matter; CP—crude protein; EE—ether extractives; CF—crude fiber; TPC—total phenol content; TAC—total antioxidant capacity; FAME—fatty acids methyl esters; MUFA—monounsaturated fatty acids; PUFA—polyunsaturated fatty acids; SFA—saturated fatty acids; UFA—unsaturated fatty acids.

**Table 3:** Proximate composition of breast and thigh meat samples.

Specification	C	E1	E2	SEM	*p*-Value
Breast					
DM, %	25.33	25.71	25.36	0.214	0.743
CP, %	22.79	23.18	22.98	0.192	0.801
EE, %	1.47 ^a^	1.51 ^a^	1.32 ^b^	0.024	<0.0001
Ash, %	1.05	1.07	1.05	0.010	0.820
Thigh					
DM, %	23.48	23.90	24.02	0.266	0.710
CP, %	18.85	19.22	18.96	0.206	0.778
EE, %	3.74	3.71	3.96	0.059	0.0515
Ash, %	0.86	0.89	0.90	0.012	0.364

C = control diet; E1 = experimental diet supplemented with 200 µg/kg diet CrPic; E2 = experimental diet supplemented with 200 µg/kg diet CrPic + 10 g creeping wood sorrel powder (CWS)/kg diet; DM—dry matter; CP—crude protein; EE—ether extractives; ^a,b^ Means within a row with no common superscript differ (*p* < 0.05); SEM = standard error of the mean.

**Table 4:** The fatty acid profile of breast meat.

Fatty Acids (g/100 g Fatty Acids Methyl Esters)	C	E1	E2	SEM	*p*-Value
C14:0	0.61	0.43	0.45	0.047	0.251
C15:0	0.14 ^a^	0.11 ^b^	0.11 ^a,b^	0.005	0.027
C16:0	18.15 ^b^	18.08 ^b^	18.65 ^a^	0.091	0.008
C17:0	0.21 ^a^	0.21 ^a^	0.18 ^b^	0.004	<0.0001
C18:0	7.57 ^b^	7.76 ^a,b^	8.30 ^a^	0.134	0.047
C24:0	0.26 ^a^	0.22 ^b^	0.26 ^a^	0.006	0.008
Σ SFA	26.88	26.84	27.98	0.244	0.084
C14:1	0.08 ^a^	0.07 ^a,b^	0.05 ^b^	0.005	0.046
C15:1	0.56	0.64	0.74	0.041	0.202
C16:1	2.53 ^a^	2.48 ^a^	2.17 ^b^	0.048	<0.0001
C17:1	0.13 ^b^	0.16 ^a,b^	0.20 ^a^	0.010	0.008
C18:1	29.46 ^a^	29.63 ^a^	28.48 ^b^	0.175	0.004
C22:1n9	0.09 ^a^	0.07 ^b^	0.10 ^a^	0.005	0.002
C24:1n9	1.03 ^b^	1.08 ^b^	1.37 ^a^	0.047	<0.0001
Σ MUFA	33.85 ^a^	34.11 ^a^	33.10 ^b^	0.161	0.009
C18:2n6	32.42 ^a^	32.35 ^a^	31.31 ^b^	0.189	0.013
C18:3n6	0.29 ^a^	0.30 ^a^	0.23 ^b^	0.010	<0.0001
C20:2n6	0.26	0.22	0.22	0.009	0.096
C20:3n6	0.79 ^a^	0.70 ^b^	0.82 ^a^	0.017	0.002
C20:4n6	2.64 ^b^	2.89 ^b^	3.43 ^a^	0.106	<0.0001
C22:2n6	0.16 ^b^	0.15 ^b^	0.22 ^a^	0.010	0.003
C22:3n6	0.13	0.17	0.18	0.009	0.067
C22:4n6	0.34 ^b^	0.30 ^b^	0.40 ^a^	0.012	0.001
Σ n − 6	37.26	37.08	36.81	0.117	0.311
C18:3n3	0.69 ^a^	0.59 ^b^	0.54 ^b^	0.023	0.006
C18:4n3	0.19 ^c^	0.30 ^b^	0.38 ^a^	0.024	<0.0001
C20:3n3	0.50 ^b^	0.48 ^b^	0.57 ^a^	0.013	0.007
C20:5n3	0.22 ^a^	0.17 ^b^	0.20 ^a,b^	0.007	0.005
C22:5n3	0.11	0.12	0.13	0.004	0.063
C22:6n3	0.06 ^a^	0.07 ^a,b^	0.10 ^b^	0.007	0.015
Σ n − 3	1.79 ^b^	1.74 ^b^	1.93 ^a^	0.029	0.007
Σ PUFA	39.05	38.82	38.73	0.114	0.551
Other fatty acids	0.23	0.23	0.19	0.013	0.474

C = control diet; E1 = experimental diet supplemented with 200 µg/kg diet CrPic; E2 = experimental diet supplemented with 200 µg/kg diet CrPic + 10 g creeping wood sorrel powder (CWS)/kg diet; MUFA = monounsaturated fatty acids; PUFA = polyunsaturated fatty acids; SFA = saturated fatty acids; ^a,b,c^ Means within a row with no common superscript differ (*p* < 0.05); SEM = standard error of the mean.

**Table 5:** The fatty acid profile of thigh meat.

Fatty Acids (g/100 g Fatty Acids Methyl Esters)	C	E1	E2	SEM	*p*-Value
C14:0	0.50	0.46	0.45	0.015	0.057
C15:0	0.19	0.15	0.15	0.009	0.054
C16:0	19.13	18.87	19.26	0.182	0.709
C17:0	0.22	0.24	0.23	0.003	0.326
C18:0	7.15 ^b^	7.96 ^a^	7.71 ^a^	0.096	<0.0001
C24:0	0.25	0.24	0.23	0.054	0.159
Σ SFA	27.44	27.69	27.92	0.152	0.473
C14:1	0.10	0.08	0.08	0.004	0.180
C15:1	0.91	0.83	0.70	0.045	0.159
C16:1	3.33 ^a^	2.95 ^b^	2.94 ^b^	0.055	<0.0001
C17:1	0.16	0.13	0.17	0.014	0.496
C18:1	29.59	29.42	29.33	0.084	0.444
C22:1n9	0.07	0.06	0.05	0.006	0.277
C24:1n9	0.59 ^b^	0.88 ^a^	0.81 ^a,b^	0.045	0.011
Σ MUFA	34.74 ^a^	34.36 ^a,b^	34.08 ^b^	0.099	0.012
C18:2n6	31.27	31.16	31.30	0.067	0.676
C18:3n6	0.21	0.23	0.20	0.006	0.097
C20:2n6	0.28 ^a^	0.26 ^a,b^	0.18 ^b^	0.017	0.029
C20:3n6	0.49	0.54	0.52	0.016	0.533
C20:4n6	2.82	3.10	3.03	0.061	0.151
C22:2n6	0.19	0.18	0.18	0.008	0.796
C22:3n6	0.15 ^b^	0.54 ^a^	0.14 ^b^	0.046	<0.0001
C22:4n6	0.19	0.20	0.19	0.008	0.880
Σ n − 6	35.61	35.75	35.74	0.101	0.834
C18:3n3	0.53 ^a^	0.50 ^b^	0.51 ^b^	0.004	0.003
C18:4n3	0.38 ^a^	0.32 ^a,b^	0.30 ^b^	0.013	0.038
C20:3n3	0.34	0.37	0.35	0.023	0.929
C20:5n3	0.44	0.51	0.45	0.035	0.723
C22:5n3	0.12	0.12	0.10	0.005	0.571
C22:6n3	0.08	0.05	0.06	0.005	0.094
Σ n − 3	1.85	1.85	1.83	0.042	0.981
Σ PUFA	37.55	37.60	37.53	0.131	0.979
Other fatty acids	0.36	0.48	0.21	0.048	0.073

C = control diet; E1 = experimental diet supplemented with 200 µg/kg diet CrPic; E2 = experimental diet supplemented with 200 µg/kg diet CrPic + 10 g creeping wood sorrel powder (CWS)/kg diet; MUFA = monounsaturated fatty acids; PUFA = polyunsaturated fatty acids; SFA = saturated fatty acids; ^a,b^ Means within a row with no common superscript differ (*p* < 0.05); SEM = standard error of the mean.

**Table 6:** Bioactive nutrient content of breast meat.

Bioactive Nutrient	C	E1	E2	SEM	*p*-Value
Antioxidant profile					
TPC, mg/g GAE	1.64	1.61	1.67	0.062	0.943
TAC, µM Trolox	1.81	1.99	1.98	0.044	0.177
Lutein and zeaxanthin, µg/g	2.73 ^b^	3.16 ^a,b^	3.64 ^a^	0.140	0.010
Vitamin E, µg/g	56.62 ^a^	47.56 ^b^	50.25 ^b^	1.352	0.003
Mineral profile (mg/kg)					
Copper	nd	nd	nd	-	-
Iron	28.60 ^a^	26.01 ^b^	25.03 ^b^	0.342	0.0001
Manganese	nd	nd	nd	-	-
Zinc	28.42 ^a^	26.18 ^b^	27.76 ^a^	0.329	0.003

C = control diet; E1 = experimental diet supplemented with 200 µg/kg diet CrPic; E2 = experimental diet supplemented with 200 µg/kg diet CrPic + 10 g creeping wood sorrel powder (CWS)/kg diet; TPC—total phenol content; TAC—total antioxidant capacity; nd—not detected. ^a,b^ Means within a row with no common superscript differ (*p* < 0.05); SEM = standard error of the mean.

**Table 7:** Bioactive nutrient content of thigh meat.

Bioactive Nutrient	C	E1	E2	SEM	*p*-Value
Antioxidant profile					
TPC, mg/g GAE	1.20	1.23	1.29	0.023	0.284
TAC, µM Trolox	1.98	2.00	2.05	0.062	0.897
Lutein and zeaxanthin, µg/g	1.45 ^b^	1.69 ^a,b^	1.87 ^a^	0.068	0.027
Vitamin E, µg/g	161.11 ^a^	146.48 ^a,b^	138.79 ^b^	3.878	0.037
Mineral profile (mg/kg)					
Copper	2.31	2.04	2.07	0.047	0.0761
Iron	36.18 ^b^	38.14 ^a^	35.69 ^b^	0.298	0.0135
Manganese	0.38	0.58	0.36	0.034	0.1161
Zinc	69.96 ^b^	68.58 ^c^	73.35 ^a^	0.412	<0.0001

C = control diet; E1 = experimental diet supplemented with 200 µg/kg diet CrPic; E2 = experimental diet supplemented with 200 µg/kg diet CrPic + 10 g creeping wood sorrel powder (CWS)/kg diet; TPC—total phenol content; TAC—total antioxidant capacity; nd—not detected. ^a,b,c^ Means within a row with no common superscript differ (*p* < 0.05); SEM = standard error of the mean.

## Data Availability

All data is contained within the article.
